# Evidence for converging pathophysiology in complex regional pain-syndrome and primary headache disorders: results from a case–control study

**DOI:** 10.1007/s00415-023-12119-w

**Published:** 2023-12-09

**Authors:** Matthias Wiemann, Nikolas Zimowski, Sarah-Luis Blendow, Elena Enax-Krumova, Steffen Naegel, Robert Fleischmann, Sebastian Strauss

**Affiliations:** 1 Department of Neurology, University Medicine Greifswald, Greifswald, Germany; 2 Department of Trauma, Reconstructive Surgery and Rehabilitative Medicine, University Medicine Greifswald, Greifswald, Germany; 3 Department of Neurology, BG University Hospital Bergmannsheil gGmbH, Ruhr University Bochum, Bochum, Germany; 4 Department of Neurology, Martin Luther University Halle-Wittenberg and University Hospital Halle, Halle (Saale), Germany; 5 Department of Neurology, Alfried Krupp Hospital, Essen, Germany

**Keywords:** Migraine, CRPS, Complex regional pain syndrome, Central sensitization, CGRP

## Abstract

**Background:**

Neuroinflammation and maladaptive neuroplasticity play pivotal roles in migraine (MIG), trigeminal autonomic cephalalgias (TAC), and complex regional pain syndrome (CRPS). Notably, CRPS shares connections with calcitonin gene-related peptide (CGRP) in its pathophysiology. This study aims to assess if the documented links between CRPS and MIG/TAC in literature align with clinical phenotypes and disease progressions. This assessment may bolster the hypothesis of shared pathophysiological mechanisms.

**Methods:**

Patients with CRPS (*n* = 184) and an age-/gender-matched control group with trauma but without CRPS (*n* = 148) participated in this case–control study. Participant answered well-established questionnaires for the definition of CRPS symptoms, any headache complaints, headache entity, and clinical management.

**Results:**

Patients with CRPS were significantly more likely to suffer from migraine (OR: 3.23, 95% CI 1.82–5.85), TAC (OR: 8.07, 95% CI 1.33–154.79), or non-classified headaches (OR: 3.68, 95% CI 1.88–7.49) compared to the control group. Patients with MIG/TAC developed CRPS earlier in life (37.2 ± 11.1 vs 46.8 ± 13.5 years), had more often a central CRPS phenotype (60.6% vs. 37.0% overall) and were three times more likely to report allodynia compared to CRPS patients with other types of headaches. Additionally, these patients experienced higher pain levels and more severe CRPS, which intensified with an increasing number of headache days. Patients receiving monoclonal antibody treatment targeting the CGRP pathway for headaches reported positive effects on CRPS symptoms.

**Conclusion:**

This study identified clinically relevant associations of MIG/TAC and CRPS not explained by chance. Further longitudinal investigations exploring potentially mutual pathomechanisms may improve the clinical management of both CRPS and primary headache disorders.

**Trial registration:**

German Clinical Trials Register (DRKS00022961).

**Supplementary Information:**

The online version contains supplementary material available at 10.1007/s00415-023-12119-w.

## Introduction

Chronic pain has a huge impact on patients’ quality of life, daily living, psychosocial health and poses a remarkable economic burden [1]. Different pain conditions seem to occur coincidentally, the exact nature of which remains to be explored [22]. Overlap in underlying pathomechanisms still needs to be distinguished from possibly incidental comorbidities. Trigeminal autonomic cephalalgias (TAC) and migraine represent primary headache disorders with partially overlapping pathophysiology, similar clinical features (e.g., chronobiology, cranial autonomic symptoms, cutaneous allodynia), response to calcitonin gene-related peptide (CGRP)-based therapeutics and central maladaptive changes in brain regions engaged in central sensitization (e.g., hypothalamus, insular and sensorimotor cortex) which is particularly evident in chronic forms of those headaches [4, 12, 23, 27]. The complex regional pain syndrome (CRPS), which is characterized by excruciating pain in combination with impeding sensorimotor dysfunction, involves mechanism of peripheral and central sensitization and is associated with increased levels of inflammatory neuropeptides, including CGRP [2, 26]. In line with this, CRPS patients have been reported to be three times more likely to suffer from migraine; the authors concluded that susceptibility for migraine predisposes for the development of CRPS [24]. While this hypothesis is intriguing, the association found between CRPS and migraine has not been validated in an independent cohort and the specificity of findings including other primary headache disorders is also still unknown. Furthermore, interactions of the clinical course and therapeutic response need to be demonstrated to implicate shared pathophysiology [16].

The present study aims to fill this gap and examine comprehensively several hypotheses (H) related to a possible association between primary headache disorders and CRPS in terms of clinical phenotypes and treatment response. We hypothesized that migraine and TAC, but not tension-type headache, are more prevalent in patients with CRPS than in our cohort of patients after trauma without CRPS and the expected prevalence in a general population (primary endpoint; H1). As secondary hypotheses, we assume that CRPS occurs in those patients at earlier age (H2) and that they show more often a central phenotype of CRPS according to the classification of Dimova et al. (H3) [7]. We furthermore hypothesize that CRPS severity, occurrence of anxiety and depression and quality of life differs between patients CRPS with and without concomitant migraine/TAC (H4). Given that central sensitization is a widely acknowledged characteristic of migraine, especially in cases of chronic migraine [12], we also analyzed any association between headache frequency and the clinical features mentioned above in the subgroup of migraine patients, and assumed statistically higher severity in CRPS patients suffering concomitant chronic migraine compared to episodic migraine. Finally, we expect a positive effect of CGRP monoclonal antibody (mAb) prevention for migraine on CRPS-related symptoms (H5) [2]

## Methods

### Study design and registration, patient recruitment

Patients suffering from CRPS were prospectively recruited for a cross-sectional survey through specialized pain centers and by making contact via patient support groups between 03/2020 and 09/2021. The diagnosis had to be either confirmed by the treating pain center, or patients recruited through support groups had to specify place and date of diagnosis, as well as the triggering event. Further, the International Association for the Study of Pain (IASP) diagnostic criteria for CRPS were also assessed in the medical history [14], and reviewed before study inclusion. Further inclusion criteria were an existing CRPS diagnosis of the lower or upper extremity and an age between 18 and 70 years.

As a control group, we recruited subjects from the local trauma centers between 01/2023 and 03/2023 who suffered trauma in the past but did not subsequently develop CRPS. Inclusion criteria were fracture or invasive intervention of the lower or upper limbs and age between 18 and 70 years.

**Fig. 1:**
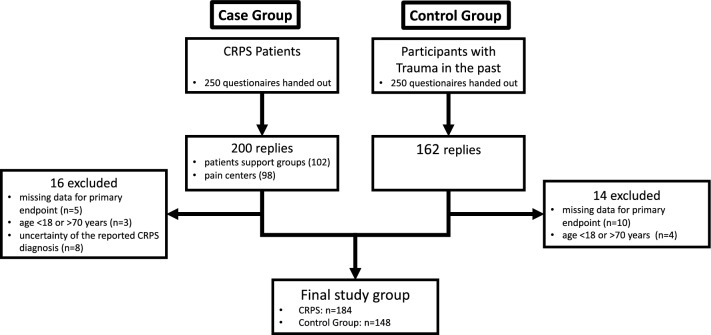
Flowchart of the included participants in the study

After handing out 250 questionnaires each, we received 200 questionnaires from CRPS patients (*n* = 102 from support groups, *n* = 98 from specialized pain centers) and 162 from patients with trauma who did not develop CRPS, which were screened for completeness of the data and inclusion criteria (Fig. 1).

### Primary endpoint assessment—prevalence of headache phenotypes

Participants were first asked whether they suffered any headache during the past year. Positive cases proceeded with a headache screening tool validated for population research (sensitivity/specificity of 72%/95% for monodiagnoses), including twenty-four questions and ten sub-questions based on international classification of headache disorders criteria (ICHD) [13]. Depending on the screening results, patients were classified to suffer from migraine (MIG), tension-type headache (TTH), and/or trigeminal autonomic cephalalgia (TAC). Population-based normative data for exactly the same screening tool are available and were used to compare the prevalence in the CRPS and control group to an unselected German population [28].

### Secondary endpoint assessment

#### Headache characteristics

Participants with headache were asked to answer more detailed questions about their headache history, disease duration (years), number of headache days/month (≥ 15 day/month was classified as chronic headache), duration of single headache attacks (hours) and number of days/months with abortive headache medication. Participants were asked whether the manifestation of the headache disorder preceded the diagnosis of CRPS or the trauma, respectively. In addition, the effects of abortive and preventive headache medication on different headache (“good”; “moderate”; “no” effect) and CRPS symptoms (“positive”; “no” effect) were inquired. A structured questionnaire was used to assess the impact of headache on daily activities (Headache Impact Test (HIT-6) [18].

#### CRPS characteristics

We inquired the occurrence of typical CRPS symptoms and medical history including the side of the affected limb, the triggering event, and the date of the event, the place of diagnosis and previous treatments (pharmacological and non-pharmacological). Clinical characteristics of CRPS were queried according the current IASP diagnostic criteria and were applied to further enhance the reliability of the diagnosis [14]. A visual analogue scale (VAS, 10 cm) assessed current movement pain and pain at rest. Disease severity (including questions on sensory, vaso-/sudomotor, motor and trophic dysfunction) was evaluated using an adaptation (only self-reported symptoms) of the CRPS severity score (CSS), as a validated approach [15]. Classification of CRPS patients in a predominantly “peripheral”, “mixed” and “central” phenotype was performed based on the reported symptoms referring to a recently validated algorithm [7]. The algorithm attribute symptoms/findings such as edema, skin color changes, skin temperature changes, sweating and trophic changes, predominantly to a peripheral phenotype. Minor injury eliciting CRPS, motor signs, allodynia, and glove/stocking-like sensory deficits have been suggested to reflect a central phenotype: Patients showing aspects of both major phenotypic groups were classified as mixed phenotype according to the algorithm [7].

### Assessment of demographic characteristics and patient-reported outcome measures irrespective of pain phenotype

All patients received a standardized questionnaire to assess sociodemographic characteristics. The Hospital Anxiety and Depression Scale (HADS) was used to self-report depressive and anxiety symptoms during the past week (HADS-A/-D subscales, 7 items each, four-point Likert scale, items rated 0–3 points) [29]. Quality of life was assessed using the Euroqol (EQ) instrument, measuring health dimensions of health (5D; mobility, self-care, usual activities, pain/discomfort, anxiety/depression) on a 5-level scale (5L; range 1–5 (best to worst)) [3]. Global health was rated using the EQ-visual analogue scale (EQ-VAS; range 0–100, increasing from worst to best).

### Sample size considerations and statistics

All analyses were done using R version 4.2.2. The primary hypothesis was a higher prevalence of migraine and TAC in patients with CRPS compared to a clinical control group with trauma in the past [10, 28]. Power analyses were done based on a prevalence of migraine in German population that has been estimated to be around 17% and is estimated to be between 2 and 3.6-times higher in patients with CRPS [24, 28]. A two-sided logistic regression (*z*-test) with an assumed odds ratio of 2.8, a significance level (alpha error) of 0.05, and a power of 90% (beta error), required a group size of at least 219 participants (CRPS patients and participants without CRPS) to detect a significant difference. (G*Power version 3.1.9) [11]. Assuming a high rate (20%) of incomplete questionnaires a total of at least 275 participants had to be included.

Differences of group means of continuous (dependent) variables were analyzed using a one-way analysis of variance (ANOVA) and Tukey’s post-hoc test. When prerequisites for an ANOVA were not met (i.e., heteroscedasticity, non-normal distribution), we used a Kruskal–Wallis rank sum test with a post-hoc Dunn’s test and Holm–Bonferroni correction instead. When comparing two groups, we used Student’s *t*-test or Wilcoxon rank sum test depending on the distribution. For categorical data and frequencies, we used Chi-squared test or Fisher’s exact test and post-hoc Chi-squared test with Holm-Bonferroni correction. We used binary logistic regression to calculate the odds ratio and ninety-five percent confidence intervals (OR, 95% CI) and Pearson correlation coefficient for bivariate correlations.

To compare findings with previous studies, we calculated the age and gender-adjusted standardized morbidity ratios (SMR) with ninety-five percent confidence intervals through mid-P exact tests in analogy to Peterlin et al. 2010 [24].

The SMR reflects the amount of excess of primary headache disorders that is present in a patient population compared with the general population.

## Results

The total study population included in the statistical analysis consisted of 332 participants (231 female, mean age 47.2 ± 12.0 years). There was no significant difference in age (*p* = 0.61) or sex (*p* = 0.38) between the CRPS (*n* = 184) and control group (*n* = 148). CRPS characteristics and headache prevalence did not differ between CRPS patients recruited from support groups and CRPS patients recruited from specialized pain centers. The mixed (*n* = 91) and central (*n* = 70) phenotypes were more common than the peripheral (*n* = 25) phenotype. Further sociodemographic, headache and disease characteristics of the study population are summarized in Table 1 and elaborated in more detail in the following paragraphs.

**Table 1:** Patient’s demographics and clinical characteristics

	CRPS	Control	*p* value
*n*	184	148	
Age (mean ± (SD))	46.96 (12.07)	47.65 (11.96)	0.605
Sex, *n* (%)			0.380
m	49 (26.6)	47 (31.8)	
f	131 (71.2)	100 (67.6)	
NA	4 (2.2)	1 (0.7)	
Age at CRPS onset (Mean ± (SD))	42.11 (12.89)		
MHD ((Mean ± (SD))	12.39 (7.73)	5.72 (4.94)	< 0.001
HIT-6 score (Mean ± (SD))	62.48 (7.45)	53.88 (7.94)	< 0.001
Headache onset before CRPS onset/trauma, *n* (%)			< 0.001
No	68 (52.7)	7 (9.6)	
Yes	61 (47.3)	66 (90.4)	
Month since CRPS onset/trauma (Mean ± (SD))	56.58 (53.31)	52.77 (54.28)	0.524
CSS (Mean ± (SD))	6.39 (1.61)		
Movement pain, VAS (Mean ± (SD))	8.91 (4.86)	4.49 (3.76)	< 0.001
Resting pain, VAS (Mean ± (SD))	8.68 (4.48)	4.69 (3.83)	< 0.001
Self-perceived health, EQ-VAS (Mean ± (SD))	47.33 (20.46)	79.35 (15.23)	< 0.001
HADS Depression Score (Mean ± (SD))	8.91 (4.86)	4.49 (3.76)	< 0.001
HADS Anxiety Score (Mean ± (SD))	8.68 (4.48)	4.69 (3.83)	< 0.001
Limb, *n* (%)			< 0.001
Upper	112 (60.9)	105 (70.9)	
Lower	64 (34.8)	37 (25.0)	
Both	8 (4.3)	0 (0.0)	
NA	0 (0.0)	6 (4.1)	
Headache diagnosis, *n* (%)			< 0.001
No headache	55 (29.9)	74 (50.0)	
MIG	60 (32.6)	25 (16.9)	
TTH	12 (6.5)	22 (14.9)	
TAC	6 (3.3)	1 (0.7)	
Mixed	20 (10.9)	17 (11.5)	
Non-classified	31 (16.8)	9 (6.1)	

*p* values correspond to Wilcoxon rank-sum test for differences between participants with and without CRPS for monthly headache days, movement, and resting pain, HADS depression, and anxiety score, and self-perceived healthscale. All other *p* values correspond to Student’s *t*-test. Difference of distribution was tested with Fisher’s exact test

*n* number, *SD* standard deviation, *m* male, *f* female, *VAS* visual analogue scale, *MHD* monthly headache days, *HIT-6* Headache impact test, *CSS* CRPS severity score, *HADS* Hospital anxiety and depression scale, *EQ-VAS* EuroQol visual analog scale, *MIG* migraine, *TTH* tension-type headache, *TAC* trigeminal autonomic cephalalgias

### Headache characteristics and comparison between patients with CRPS and a control group (H1)

Seventy percent of CRPS patients (*n* = 129) experienced headaches at least once in the last year. Based on the questionnaire, 32% (*n* = 60) of the patients were classified to suffer from migraine (58% episodic), 7% (*n* = 12) to tension type headache (80% episodic) and 3% (*n* = 6) to TAC (50% < 15 d/m). A single diagnosis could not be made in twenty patients (MIG/TTH: 6% (*n* = 11); MIG/TAC 4% (*n* = 7), TTH/TAC: 1% (*n* = 2)) and in 31 patients, headache characteristic did not correspond to any of these primary headache diagnoses (17%, non-classified”).

Compared to the control group, CRPS patients were more likely to suffer from migraine (OR: 3.23, 95% CI 1.82–5.85), TAC (OR: 8.07, 95% CI 1.33–154.79), or non-classified headaches (OR: 3.68, 95% CI 1.88–7.49) and reported significant higher burden of headache as indicated by the HIT-6 (mean difference: 8.61, 95% CI 7.49–9.73). Patients in the migraine subgroup reported significantly more monthly headache days (12.8 ± 7.7 SD vs. 6.0 ± 4.4 SD, *p* = 0.002), and consequently, they also exhibited a higher proportion of chronic migraine compared to the control subjects (42.1% vs. 7.1%, *p* = 0.014).

Significant differences in the same direction were present when comparing sex and age-adjusted data to normative data from the German Headache Consortium Study, which used the same questionnaire (migraine SMR: 1.71, 95% CI 1.32–2.19; TAC SMR: 21.74, 95% CI 8.81–45.21; non-classified SMR: 1.62, 95% CI 1.18–2.18). In addition, CRPS patients were more likely to have experienced any kind of headache (SMR: 1.21, 95% CI 1.01–1.43) and less likely to have TTH (SMR: 0.52, 95% CI 0.28–0.89) or mixed migraine and TTH (SMR: 0.45, 95% CI 0.23–0.80). The control group did not significantly differ from population data, after adjusting for sex and age, see Table 2. Further details on headache characteristics can be found in the supplementary material.

**Table 2:** Prevalence and 95% confidence intervals of headache phenotypes in the general population and in the current CRPS patient

	General population		Control group				CRPS group				OR CRPS—control	
	Prevalence	95% CI	Prevalence	95% CI	SMR	95% CI	Prevalence	95% CI	SMR	95% CI	OR	95% CI
Any headache	59.30	58.37–60.31	50.00	42.05–57.95	0.90	0.71–1.12	70.11	63.14–76.26	**1.21**	1.01–1.43	1.35	0.95–1.91
MIG	17.90	17.1–18.6	16.89	11.71–23.75	0.94	0.62–1.36	32.61	26.25–39.68	**1.71**	1.32–2.19	**3.23**	1.82–5.85
TTH	13.30	12.6–13.9	14.86	10.03–21.48	1.22	0.78–1.82	6.52	3.77–11.05	**0.52**	0.28–0.89	0.73	0.33–1.59
TAC	0.15	0.01–0.55	0.68	0.12–3.73	4.50	0.23–22.22	3.26	1.5–6.93	**21.74**	8.81–45.21	**8.07**	1.33–154.79
MIG/TTH	12.80	12.2–13.5	7.43	4.2–12.82	0.64	0.33–1.10	5.43	2.98–9.71	**0.45**	0.23–0.80	1.22	0.48–3.10
Non-classified	15.30	14.6–16.0	10.14	6.24–16.05	0.76	0.44–1.22	22.28	16.87–28.83	**1.62**	1.18–2.18	**3.68**	1.88–7.49

German population data from Yoon et al. (2012). The standardized morbidity ratio reflects the amount of excess of primary headache disorders that is present in a study population compared with the general population adjusted for age and gender. Trigeminal autonomic cephalalgias (TAC) is not adjusted due to missing information of age and gender distribution in the general population

Bold numbers indicate *p* < 0.05

*CRPS* complex regional pain syndrome, *MIG* migraine, *TTH* tension type headache

### Relationship of headache and disease onset of CRPS (H2)

Based on pathophysiological considerations and insufficient statistical power of the TAC group, headache phenotypes were grouped as MIG/TAC, mixed/unclassifiable, TTH and no headache for further analyses.

**Fig. 2:**
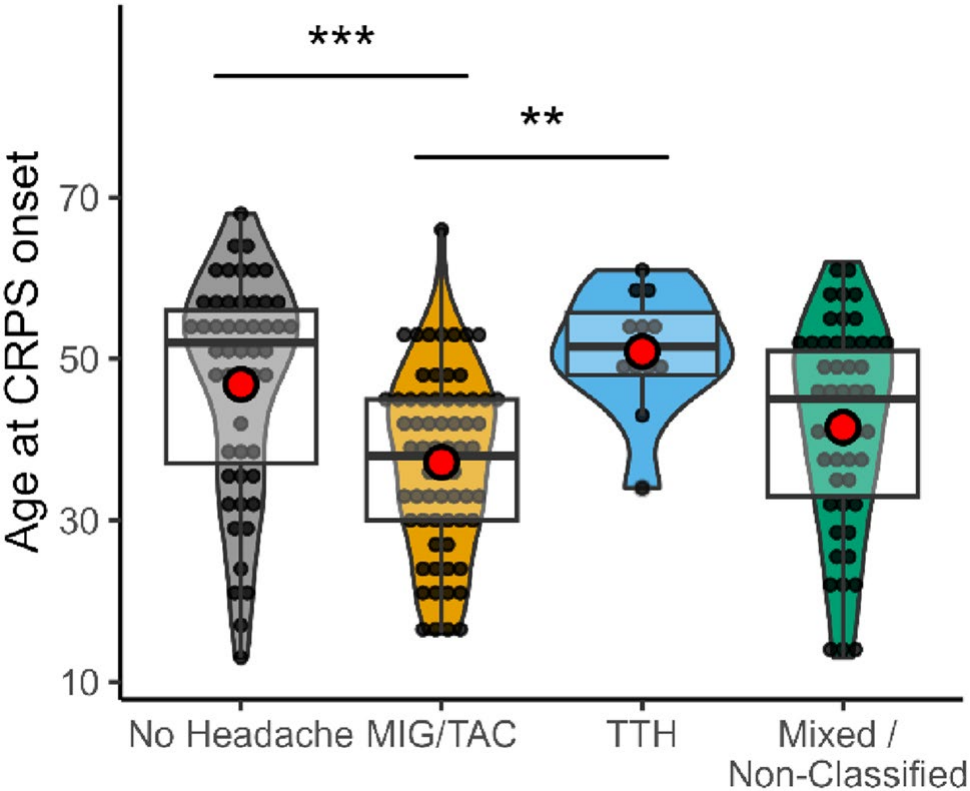
Violin plot with boxplot for age at CRPS onset dependent on headache diagnosis. Red dot indicates mean. *MIG/TAC* combined group of patients with migraine and trigeminal autonomic cephalalgias, *TTH* tension type headache, **Indicate *p* < 0.01, *** indicate *p* < 0.001

ANOVA revealed a significant association of headache phenotype and age at CRPS onset (*F*(3,173) = 8.24, *p* < 0.001). Post-hoc test yielded significantly younger age at CRPS onset in patients suffering MIG/TAC than patients without headache (37.2 ± 11.1 SD vs. 46.9 ± 13.4 SD years; − 9.6 years, 95% CI − 5.1 to − 14.2)) and patients with TTH (50.9 ± 7.5 SD years, *p* = 0.002; − 13.7, 95% CI 9.9–17.5). (Fig. 2).

### Patients with migraine and TAC show more often a central phenotype of CRPS (H3).

Chi-squared test resulted in significant differences between headache and CRPS phenotypes (chi-squared(6) = 36.4, *p* < 0.001).

Post-hoc analyses showed significantly more central CRPS phenotypes for patients with MIG/TAC (60.6% vs. 37.0% overall, *p* < 0.001) and less mixed CRPS phenotypes (25.8% vs. 49.5% overall, *p* < 0.001). Patients with mixed or non-classified headache diagnosis were more likely to have a mixed CRPS phenotypes (76.5% vs. 49.5% Overall, *p* < 0.001) and less often central CRPS phenotypes (11.8% vs. 37.0% Overall, *p* < 0.001).

Allodynia differed significantly between headache diagnosis (*p* = 0.045). A binomial regression analysis also revealed that allodynia was more frequent in CRPS-patients with MIG/TAC (OR: 3.45, 95% CI 1.4–9.4), but not in patients with TTH (OR: 4.85, 95% CI 0.8–92.9) or mixed or non-classified headache (OR: 2.15, 95% CI 0.9–5.7).

### CRPS severity, occurrence of anxiety and depression and quality of life differs depending on the headache status in CRPS (H4).

There was a significant association between the severity of CRPS (indicated by the adapted CSS) and headache type (chi-squared (3) = 9.57, *p* = 0.023). Dunn’s test for multiple comparisons with Holm’s correction resulted in significant differences between mixed/non-classified headache group and patients without headache (6.8 ± 1.2 vs 5.7 ± 2.1, *p* = 0.012).

**Fig. 3:**
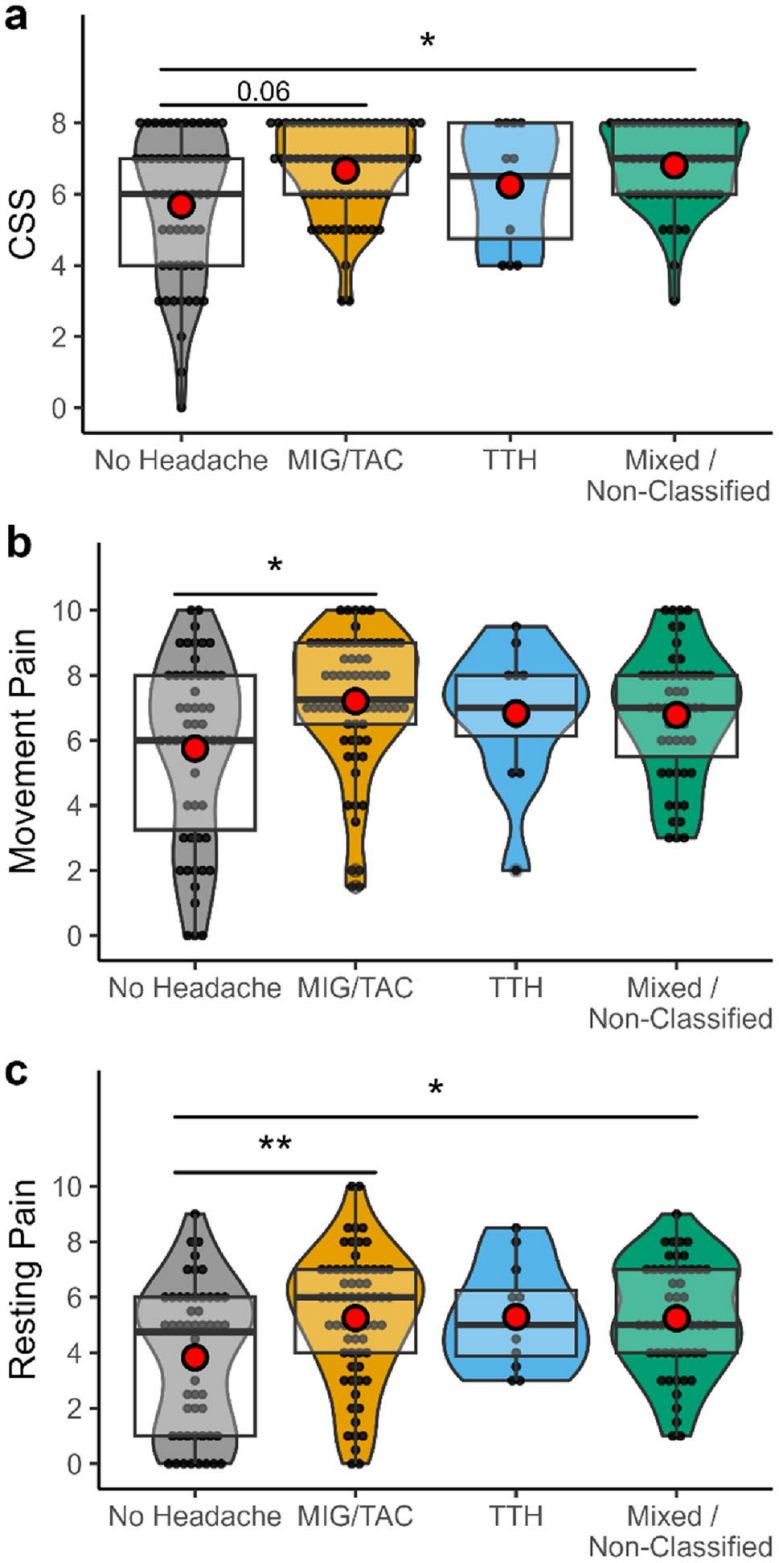
**a**–**c** Violin plot with boxplot showing CRPS severity (CSS), movement pain and resting pain dependent on headache status. Red dot indicates mean. *MIG/TAC* combined group of patients with migraine and trigeminal autonomic cephalalgias, *TTH* tension type headache. *Indicate *p* < 0.05, ** indicate *p* < 0.01

Rating of movement and resting pain differed between the groups (movement pain: chi-squared (3) = 9.57, *p* = 0.023; rest pain: *F*(3,179) = 4.78, *p* = 0.003). Patients with concomitant MIG/TAC reported significantly higher pain levels at rest (5.3 ± 2.4 vs 3.8 ± 2.6, *p* = 0.006) and on movement (7.2 ± 2.1 vs 5.8 ± 2.8, *p* = 0.01) compared to patients without headache. Patients with mixed or non-classified headaches also reported higher pain levels at rest (5.3 ± 2.0 vs. 3.8 ± 2.6, *p* = 0.01) compared to patients without headache. Results are summarized in Fig. 3a–c.

ANOVA revealed a significant association between headache phenotypes and depression (*F*(3,180) = 7.79, *p* < 0.001), anxiety (*F*(3,180) = 7.74, *p* < 0.001), the VAS (*F*(3,178) = 4.18, *p* = 0.007), and quality of life (*F*(3,169) = 6.26, *p* < 0.001). The Post-hoc test demonstrated that CRPS patients with MIG/TAC and mixed headache syndromes had a higher HADS-depression score compared to patients without headache (MIG/TAC: 10.2 ± 5.3 vs 6.5 ± 4.1, *p* < 0.001; mixed/non-classified: 10.08 ± 4.47 vs 6.5 ± 4.1, *p* < 0.001). In these patients HADS-anxiety score was also higher compared to patients without headache (MIG/TAC: 9.9 ± 4.7 vs 6.4 ± 3.4, *p* < 0.001; mixed/non-classified: 9.5 ± 4.3 vs 6.4 ± 3.4, *p* = 0.001).

**Fig. 4:**
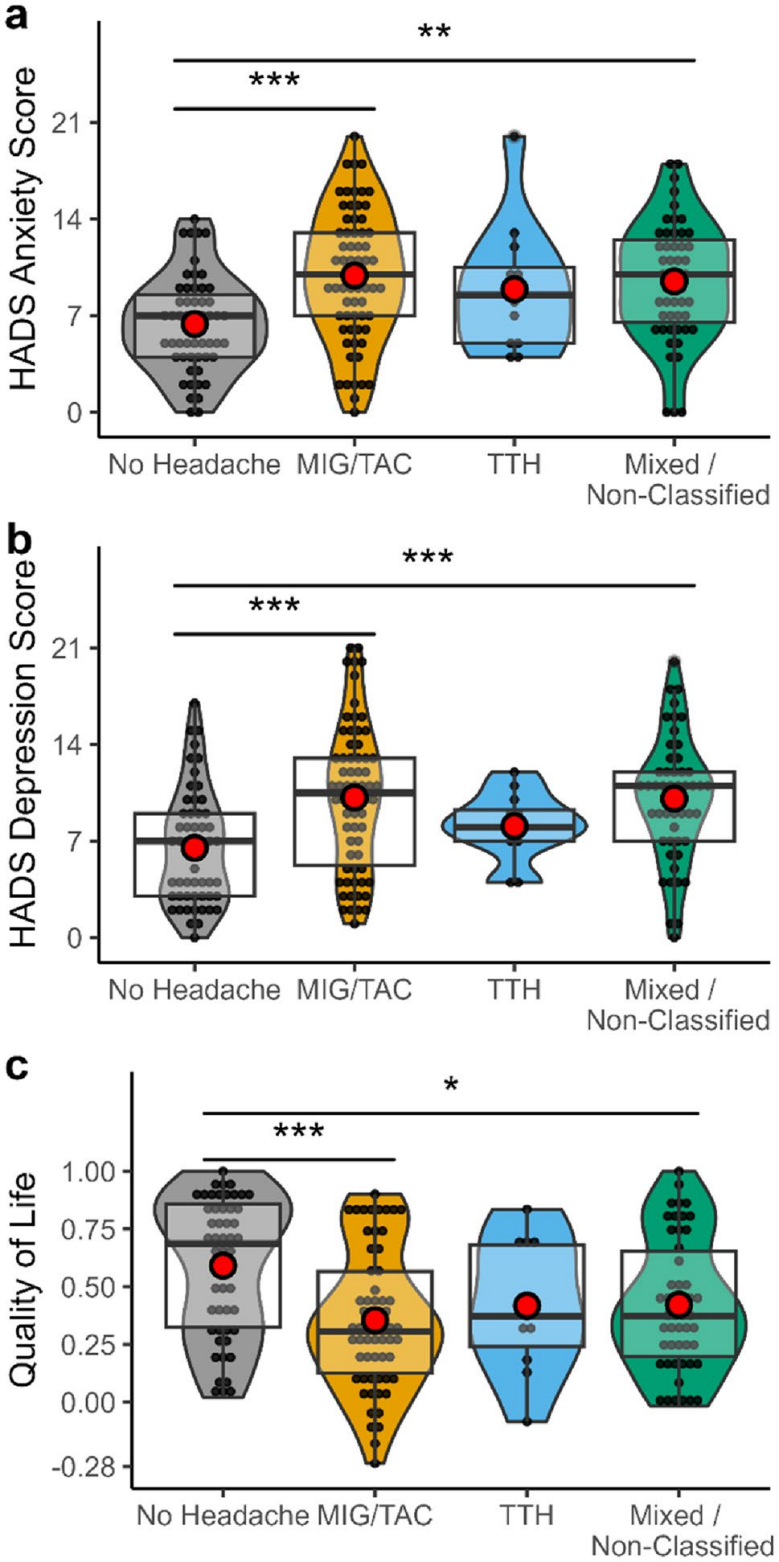
**a**–**c** Violin plot with boxplot showing occurrence of anxiety, depression and quality of life depending on headache status. Higher values in The Hospital Anxiety and Depression Scale (HADS) and in EQ-5D-5L indicating more severe anxiety, depression, and better quality of life respectively. Red dot indicates mean. *MIG/TAC* combined group of patients with migraine and trigeminal autonomic cephalalgias, *TTH* tension type headache. *Indicate *p* < 0.05, ** indicate *p* < 0.01, *** indicate *p* < 0.001

Patients with MIG/TAC also rated their individual health status significantly lower than patients without headache (43.6 ± 21.5 vs 55.2 ± 20.5, *p* = 0.009) and reported poorer quality of life (0.35 ± 0.30 vs 0.59 ± 0.30, *p* < 0.001). Quality of life was also lower in patients with mixed/non-classified headaches (0.42 ± 0.28 vs 0.59 ± 0.30, *p* = 0.022) than patients without concomitant headache (Fig. 4a–c).

**Fig. 5:**
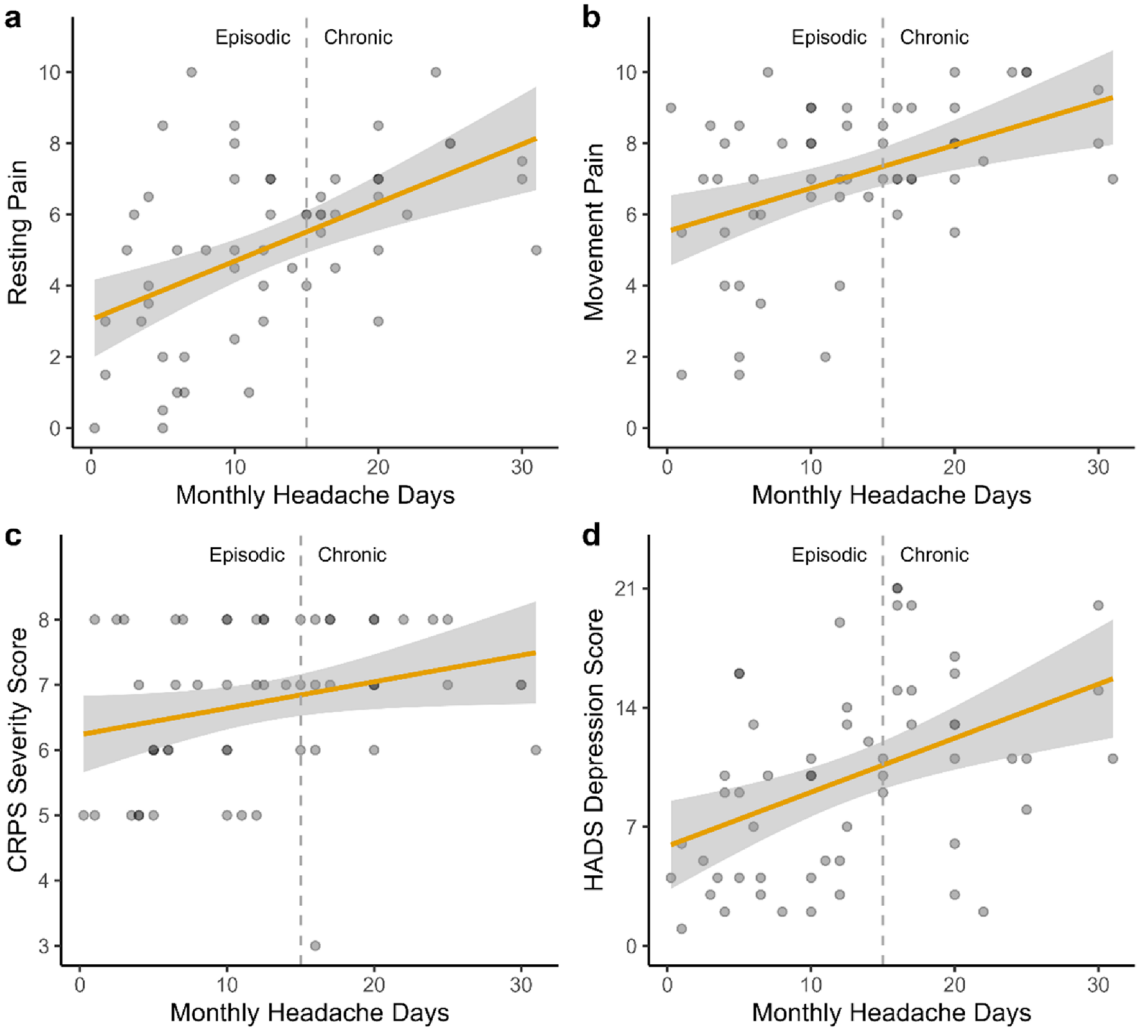
**a**–**d** Dot plots with regression line showing correlation of monthly headache days with resting pain, movement pain, CRPS severity, and depression scores in the subgroup of CRPS patients with migraine. Higher values in The Hospital Anxiety and Depression Scale (HADS) indicate more severe depression. Orange line indicates direction of correlation with grey areas around it representing its 95% confidence interval. The vertical dashed line represents the division between episodic headache (less than 15 monthly headache days) and chronic headache (15 or more monthly headache days). Darker dots represent multiple entries with the same values

When analyzing the patient subgroup with migraine we found a strong correlation of MHD with resting pain (*r* = 0.52, 95% CI 0.23–0.68), a moderate correlation with movement pain (*r* = 0.44, 95% CI 0.20–0.63), and HADS depression score (*r* = 0.44, 95% CI 0.20–0.63), as well as a small correlation with CSS (*r* = 0.27, 95% CI 0.01–0.49). There was no significant correlation between MHD and HADS anxiety score (*r* = 0.22, 95% CI − 0.05 to 0.45) or HIT-6 score (*r* = 0.06, 95% CI − 0.20 to 0.31). (Fig. 5a–d). In addition, it is noteworthy that almost all CRPS patients with chronic migraine (*n* = 22, one “not sure”) reported allodynia.

### Effect of therapeutic headache treatment on CRPS (H5)

About twenty percent (*n* = 16) of participants reported either previous or current use of preventive medication for their headaches. Seven patients used CGRP mAbs for migraine prevention, and six of which indicated a good therapeutic effect on headache symptoms whereas one patient reported no effect. All patients reported a positive effect of CGRP mAbs on CRPS symptoms.

Five patients reported a current or previous intake of topiramate as a preventive therapy (good effect: *n* = 3, moderate: *n* = 1; no effect: *n* = 1). One of these reported a beneficial effect on CRPS due to topiramate. Three patients reported intake of flunarizine or/and metoprolol/bisoprolol and reported moderate effects on headache and lack of effect on CRPS.

## Discussion

The present national cohort of CRPS patients provides evidence not only of a higher prevalence and severity of headache compared to subjects after trauma without CRPS, but also of a shift in prevalence of the different primary headache syndromes. Patients suffering coincidentally from migraine and TAC, developed CRPS earlier in life, presented rather with a “central” CRPS phenotype and reported more frequently allodynia. Thus, our results support previous findings of an association between migraine and CRPS [24], indicate probably shared pathophysiological mechanisms and provide first evidence of possible new therapeutic targets in CRPS.

### Headache prevalence in CRPS depends on type of primary headache

The prevalence of both migraine and TAC was higher in our CRPS cohort compared to a control group without CRPS and to reference data from the German population. The control group did not differ in headache prevalence and type compared to the normal population. The higher prevalence of migraine in patients with CRPS is well in line with a previous study, reporting migraine in 63% of the CRPS patients [24]. Expanding previous studies, which focused on migraine, we used a validated screening tool being able to distinguish between MIG, TTH and TAC [13]. Differences in data evaluation might explain the diverging prevalence of headache in general and MIG between our data and pre-existing literature (peterlin: 63%; in our study: 33%). Further, differences in the clinical characteristics of the CPRS cohort might also contribute to the different results (duration of CRPS: peterlin: mean 9.1 years; our cohort: 4.7 years; age of CRPS onset peterlin: 35.1 years; our cohort: 42.1 years; number of affected limbs (≥ 2; peterlin: 72%, our cohort: 4%).

### The role of central sensitization in CRPS and migraine/ TACs

Since migraine and TACs share partly common pathophysiology [4, 8, 23] the higher prevalence of both primary headache syndromes in CRPS is not surprising. These results further indicate dysfunctional habituation to sensory stimuli and mechanisms of peripheral and central sensitization not only in primary headache syndromes but also in CRPS. Central sensitization in general is known to be associated with abnormal neuronal excitability in the trigeminal brainstem nuclear complex and is thought to be clinical expressed by cutaneous hypersensitivity and allodynia [8]. Accordingly, allodynia was reported in 77% of our patients with CRPS and was more common in patients with concomitant migraine or TAC. The latter group also predominantly showed symptoms of central maladaptive reorganization and could be classified as patients with a central phenotype [7]. Interestingly, patients with migraine or TAC developed CRPS earlier in life so that it could be assumed that preexisting vulnerability may be a bidirectional risk factor for the development of both migraine/TAC as well as CRPS. It is conceivable that migraine represents a 'sensitive state' characterized by signs of neurogenic inflammation, imbalanced biochemical factors, and mechanisms of central sensitization. These factors could potentially signify a preexisting vulnerability to CRPS (‘the first hit’) thus increasing the overall likelihood of developing CRPS after a trauma (the 'second hit'). However, it is important to note that this association is somehow speculative, as detailed information on the history of prior traumata before the onset of CRPS. This hypothesis aligns with existing literature and has been discussed, for instance, by Peterlin et al. [24].

Maladaptive central sensitization in CRPS has been shown to be associated with altered excitability in both sensory and motor system (for reviews see [5, 6]) and is associated with higher pain in the affected limb. We found higher reported pain levels in patients with CRPS and concomitant migraine or TAC in general, as well as an association of number of monthly headache days and severity of CRPS in the migraine subgroup. This is particularly intriguing because central sensitization is recognized as a main factor in the development of chronic migraine [12] which reinforce the shared pathophysiological mechanism and, for the first time, suggests a mutual amplification of CRPS and migraine and/or TAC.

In addition to excruciating pain, psychological aspects such as depression and anxiety are known to be relevant in both CRPS and primary headache syndromes (CRPS: [20]; Migraine: [19]; TAC: [21]). Although all patients were severely affected by CRPS, our data suggests an additional impact of migraine and TAC on depression and anxiety symptoms, as well as individual health perception and reported quality of life. Therefore, these aspects need to be considered even more extensively for future treatment concepts.

### CGRP-based therapies as a potential novel treatment for CRPS

Finally, our survey revealed the very first anecdotal evidence for CGRP as a potential therapeutic target, not only in migraine and cluster headache, but also in CRPS, similar to the recently reported case series of patients with chronic migraine and peripheral neuropathic pain [17]. The role of CGRP and other neuropeptides such as bradykinin and substance P, as well as elevated levels of pro-inflammatory cytokines such as TNF-α, interleukin (IL)-1b, IL-2, and IL-6 has been discussed in pathophysiology of CRPS [2, 9]. Further studies to systematically capture CGRP levels in the course of CRPS as well as possible therapeutic effects of prophylactic headache treatment including CGRP antibodies on peripheral and central symptoms of CRPS are essential to be able to identify new innovative and individual therapeutic strategies for these patients.

### Limitations

Although reference data on migraine and TTH were assessed with the same established questionnaire [13] and validation process of the questionnaire showed good sensitivity and very high specificity in detecting migraine, TTH, and TAC (sensitivity: > 0.72; specify: > 0.95), there were no comparable data on the prevalence of TAC in Germany. Therefore, in addition to the included control group the prevalence of TAC was compared with the results of an epidemiological study on cluster headache in Germany since cluster is by far the most frequent TAC. Newer studies also estimated slightly different prevalence for TTH and migraine but could not be used as reference as different evaluation methods were used [25]. Moreover, given the substantial proportion of headaches that remain unclassified in our study, future research should contemplate employing structured interviews to ultimately establish a definitive diagnosis for headaches.

Although half of our CRPS patients were recruited directly via specialized pain centers and the relevant clinical data did not differ between those and patients recruited via patient support groups a degree of uncertainty in the diagnosis of CRPS, as well as in disease severity and clinical phenotype cannot be excluded.

Only twelve CRPS patients had concomitant TTH, making the sample too small to detect small effects. Therefore, differences between patients with TTH, other headache syndromes, or no headache need to be interpreted carefully. In general, this study was exploratory and meant to shed light on presumed shared mechanisms between primary headache disorders and CRPS. This being said, we conducted the study without major inclusion or exclusion criteria, and were using multiple ways of patient recruitment, which ultimately had two effects. First, heterogeneity was rather large as indicated by wide CIs of the found results. Second, for the first time, we provide statistically significant associations of the clinical course and phenotypes as a solid basis to motivate future research, while more accurate estimates of true effect sizes of identified associations require designated prospective investigations.

Finally, caution is required when interpreting the results since questionnaire studies are subject to sampling and assessment biases, as well as recall and volunteer bias. Again, further prospective studies with thorough clinical examinations, standardized symptom assessment such as e.g., quantitative sensory testing and neurophysiological measurements are needed to clarify both the probably shared pathophysiology and possible novel treatment strategies.

## Conclusions

This case–control study identified a systematic overlap between headache disorders and CRPS that cannot be explained by chance and is clinically relevant. This provides the intriguing perspective to use insights from research and clinical management for mutual benefits of both conditions. In particular, the efficacy of CGRP mAbs deserves further investigation.

### Supplementary Information

Below is the link to the electronic supplementary material.

Supplementary file1 (DOCX 88 KB)

## Data Availability

The datasets used and/or analyzed during the current study are available from the corresponding author on reasonable request.
