# Supplementary material for: Evidence for converging pathophysiology in complex regional pain-syndrome and primary headache disorders: results from a case–control study
Source: J Neurol. 2023 Dec 9;271(4):1850–60. doi: 10.1007/s00415-023-12119-w (PMC10972976; doi:10.1007/s00415-023-12119-w)
Supplement: Supplementary file 1 — Supplementary file1 (DOCX 88 KB) [file 415_2023_12119_MOESM1_ESM.docx]

Evidence for converging pathophysiology in complex regional pain-syndrome and primary headache disorders – results from a case-control study

Matthias Wiemann^1^*, Nikolas Zimowski^2^*, Sarah-Luis Blendow^1^, Elena Enax-Krumova^3^, Steffen Naegel^4,5^, Robert Fleischmann^1^, Sebastian Strauss^1^

* equal contribution

*1 Department of Neurology, University Medicine Greifswald, Greifswald, Germany*

*2 Department of Trauma, Reconstructive Surgery and Rehabilitative Medicine, University Medicine Greifswald, Greifswald, Germany*

*3 Department of Neurology, BG University Hospital Bergmannsheil GmbH, Ruhr University Bochum, Bochum, Germany*

*4 Department of Neurology, Martin Luther University Halle-Wittenberg and University Hospital Halle, Halle (Saale), Germany*

*5 Department of Neurology, Alfried Krupp Hospital, Essen, Germany*

Matching of control and CRPS group


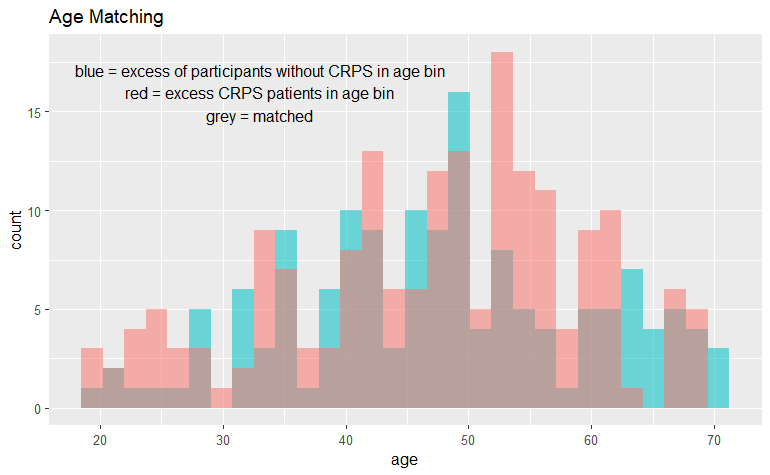


Supplementary Figure 1: Overlap of participants age between CRPS patients and participants in the control group.


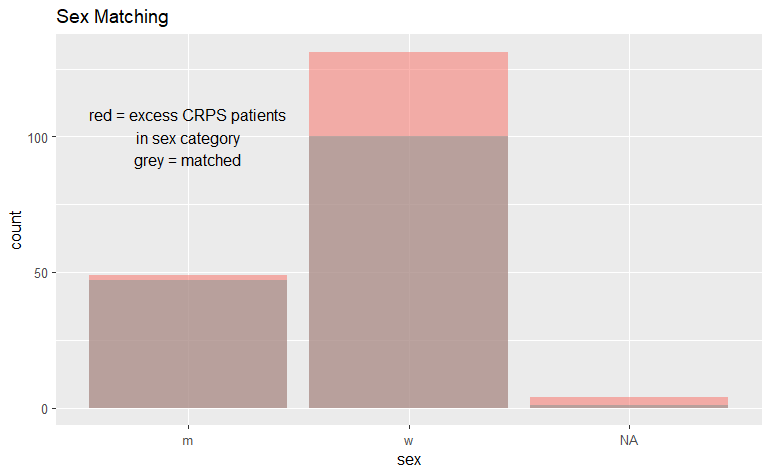


Supplementary Figure 2: Overlap of participants sex between CRPS patients and participants in the control group.

Headache Characteristics

|  | Overall | CRPS | Control | p-value |
| --- | --- | --- | --- | --- |
| n | 203 | 129 | 74 |  |
| Headache diagnosis, n (%) |  |  |  | 0.001 |
| MIG | 85 (42.1) | 60 (46.5) | 25 (33.8) |  |
| TTH | 34 (16.8) | 12 (9.3) | 22 (29.7) |  |
| TAC | 7 (3.5) | 6 (4.7) | 1 (1.4) |  |
| Mixed | 37 (18.3) | 20 (15.5) | 17 (23.0) |  |
| Non-Classified | 39 (19.3) | 31 (24.0) | 9 (12.2) |  |
| Years with hedache (mean ± (SD)) | 15.67 (12.32) | 14.06 (13.47) | 18.08 (9.95) | 0.002 |
| HIT-6 Score (mean ± (SD)) | 59.33 (8.67) | 62.48 (7.45) | 53.88 (7.94) | <0.001 |
| Monthly Headache Days (mean ± (SD)) | 10.17 (7.59) | 12.39 (7.73) | 5.72 (4.94) | <0.001 |
| Chronic Headache, n (%) | 53 (32.9) | 47 (39.5) | 6 (10.0) | 0.002 |
| Headache attack duration in hours (mean ± (SD)) | 16.52 (19.35) | 18.84 (20.83) | 12.45 (15.79) | 0.112 |
| Any medication intake against headache, n (%) |  |  |  | 0.027 |
| no | 9 (5.3) | 9 (8.3) | 0 ( 0.0) |  |
| yes | 162 (94.7) | 100 (91.7) | 62 (100.0) |  |
| NA | 32 | 20 | 12 |  |
| Headache medication intake in days/month (mean ± (SD)) | 8.96 (10.53) | 12.22 (11.78) | 3.59 (4.36) | <0.001 |
| Good | 27 (64.3) | 21 (70.0) | 6 (50.0) |  |
| Moderate | 12 (28.6) | 8 (26.7) | 4 (33.3) |  |
| Bad | 3 (7.1) | 1 (3.3) | 2 (16.7) |  |
| Effectiveness of NSARs against headache, n (%) |  |  |  | 0.076 |
| Good | 68 (47.2) | 38 (41.3) | 30 (57.7) |  |
| Moderate | 50 (34.7) | 33 (35.9) | 17 (32.7) |  |
| Bad | 26 (18.1) | 21 (22.8) | 5 (9.6) |  |
| Any prophylaxis against headache, n (%) |  |  |  | 1.000 |
| no | 41 (82.0) | 37 (82.2) | 4 (80.0) |  |
| yes | 9 (18.0) | 8 (17.8) | 1 (20.0) |  |
| NA | 153 | 84 | 69 |  |

Supplementary Table 1: P-values corresponds to Wilcoxon signed-rank test for differences between between participants with and without CRPS for all continuous data. Difference of distribution was tested with Fisher’s exact test. n = number, SD = standard deviation.


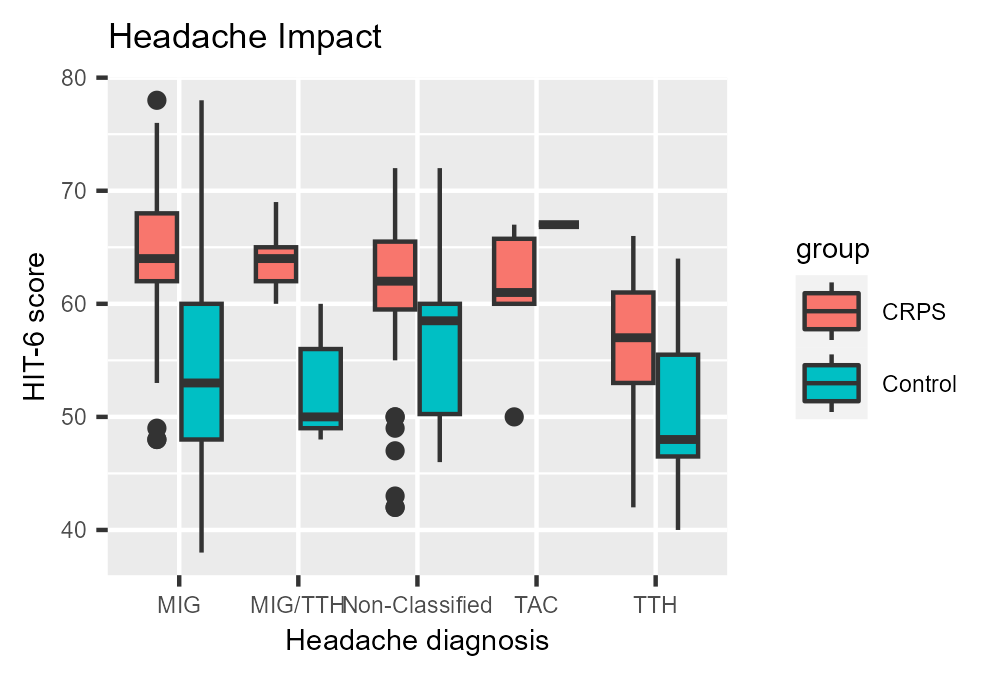


Supplementary Figure 3: Boxplots of impact of headache by headache diagnosis measured with the Headache Impact Test (HIT-6). Higher values indicate higher burden of headache.

Clinical Characteristics by gender

Male participants

|  | Overall | CRPS | Control | p-value |
| --- | --- | --- | --- | --- |
| n | 96 | 49 | 47 |  |
| Age (mean ± (SD)) | 46.47 (12.36) | 48.31 (12.34) | 44.55 (12.21) | 0.138 |
| Age at CRPS onset (mean ± (SD)) |  | 42.53 (13.56) |  |  |
| headache = Headache (%) | 42 (43.8) | 34 (69.4) | 8 (17.0) | <0.001 |
| Monthly Headache Days (mean ± (SD)) | 10.61 (8.41) | 13.65 (8.32) | 3.89 (3.00) | <0.001 |
| Chronic Headache, n (%) |  |  |  | 0.134 |
| no | 22 (62.9) | 17 (56.7) | 5 (100.0) |  |
| yes | 13 (37.1) | 13 (43.3) | 0 (0.0) |  |
| NA | 7 | 4 | 3 |  |
| HIT-6 score (mean ± (SD)) | 61.23 (7.89) | 63.00 (7.33) | 53.14 (4.88) | 0.002 |
| Headache onset before CRPS onset/trauma, n (%) |  |  |  | 0.0448 |
| No | 21 (50.0) | 20 (58.8) | 1 (12.5) |  |
| Yes | 21 (50.0) | 14 (41.2) | 7 (87.5) |  |
| NA | 54 | 15 | 39 |  |
| Month since CRPS onset/trauma (mean ± (SD)) | 62.68 (51.07) | 68.17 (57.06) | 57.19 (44.21) | 0.300 |
| CSS (mean ± (SD)) |  | 6.20 (1.79) |  |  |
| Movement pain, VAS (mean ± (SD)) |  | 6.91 (2.09) |  |  |
| Resting pain, VAS (mean ± (SD)) |  | 5.25 (2.08) |  |  |
| Limb, n (%) |  |  |  | 0.431 |
| Upper | 61 (63.5) | 29 (59.2) | 32 (68.1) |  |
| Lower | 33 (34.4) | 18 (36.7) | 15 (31.9) |  |
| Both | 2 ( 2.1) | 2 ( 4.1) | 0 ( 0.0) |  |
| Headache diagnosis, n (%) |  |  |  | <0.001 |
| No Headache | 54 (56.2) | 15 (30.6) | 39 (83.0) |  |
| MIG | 15 (15.6) | 14 (28.6) | 1 ( 2.1) |  |
| TTH | 3 ( 3.1) | 1 ( 2.0) | 2 ( 4.3) |  |
| TAC | 2 ( 2.1) | 2 ( 4.1) | 0 ( 0.0) |  |
| Mixed | 7 ( 7.3) | 4 ( 8.2) | 3 ( 6.4) |  |
| Non-Classified | 15 (15.6) | 13 (26.5) | 2 ( 4.3) |  |

Female participants

|  | Overall | CRPS | Control | p-value |
| --- | --- | --- | --- | --- |
| n | 231 | 131 | 100 |  |
| Age (mean ± (SD)) | 47.32 (11.80) | 45.97 (11.76) | 49.08 (11.68) | 0.047 |
| Age at CRPS onset (mean ± (SD)) |  | 41.51 (12.46) |  |  |
| headache = Headache (%) | 161 (69.7) | 95 (72.5) | 66 (66.0) | 0.356 |
| Monthly Headache Days (mean ± (SD)) | 9.60 (7.33) | 11.95 (7.51) | 5.79 (5.14) | <0.001 |
| Chronic Headache, n (%) |  |  |  | <0.001 |
| No | 104 (72.2) | 55 (61.8) | 49 (89.1) |  |
| Yes | 40 (27.8) | 34 (38.2) | 6 (10.9) |  |
| NA | 17 | 6 | 11 |  |
| HIT-6 score (mean ± (SD)) | 58.86 (8.82) | 62.31 (7.52) | 53.95 (8.22) | <0.001 |
| Headache onset before CRPS onset/trauma, n (%) |  |  |  | <0.001 |
| No | 54 (33.8) | 48 (50.5) | 6 ( 9.2) |  |
| Yes | 106 (66.3) | 47 (49.5) | 59 (90.8) |  |
| NA | 71 | 36 | 35 |  |
| Month since CRPS onset/trauma (mean ± (SD)) | 52.30 (54.77) | 53.41 (51.73) | 50.82 (58.83) | 0.724 |
| CSS (mean ± (SD)) |  | 6.52 (1.46) |  |  |
| Movement pain, VAS (mean ± (SD)) |  | 6.59 (2.36) |  |  |
| Resting pain, VAS (mean ± (SD)) |  | 4.74 (2.46) |  |  |
| Limb, n (%) |  |  |  | 0.0166 |
| Upper | 153 (68.0) | 81 (61.8) | 72 (76.6) |  |
| Lower | 66 (29.3) | 44 (33.6) | 22 (23.4) |  |
| Both | 6 ( 2.7) | 6 ( 4.6) | 0 ( 0.0) |  |
| NA | 6 | 0 | 6 |  |
| Headache diagnosis, n (%) |  |  |  | 0.0288 |
| No Headache | 70 (30.3) | 36 (27.5) | 34 (34.0) |  |
| MIG | 70 (30.3) | 46 (35.1) | 24 (24.0) |  |
| TTH | 31 (13.4) | 11 ( 8.4) | 20 (20.0) |  |
| TAC | 5 ( 2.2) | 4 ( 3.1) | 1 ( 1.0) |  |
| Mixed | 30 (13.0) | 16 (12.2) | 14 (14.0) |  |
| Non-Classified | 25 (10.8) | 18 (13.7) | 7 ( 7.0) |  |

Supplementary Table 2: Participants characteristics separated by gender. P-values corresponds to Student’s t-test for differences between participants with and without CRPS for all continuous data, with the exception of Monthly Headache Days, where a Wilxocon- rank sum test was performed. Difference of distribution for headache occurrence was tested with Chi-squared test. All other differences of distribution were tested with Fisher’s exact test. n = number, SD = standard deviation.
